# Supplementary material for: The interactome of intact mitochondria by cross-linking mass spectrometry provides evidence for coexisting respiratory supercomplexes
Source: Mol Cell Proteomics. 2017 Dec 8;17(2):216–32. doi: 10.1074/mcp.RA117.000470 (PMC5795388; doi:10.1074/mcp.RA117.000470)
Supplement: Supplemental Data [file supp_RA117.000470_133922_0_supp_23978_fzffwf.zip › spectra_annotation/mito_DR_spectra_annotation/200-1-18-1-26-1.pdf]

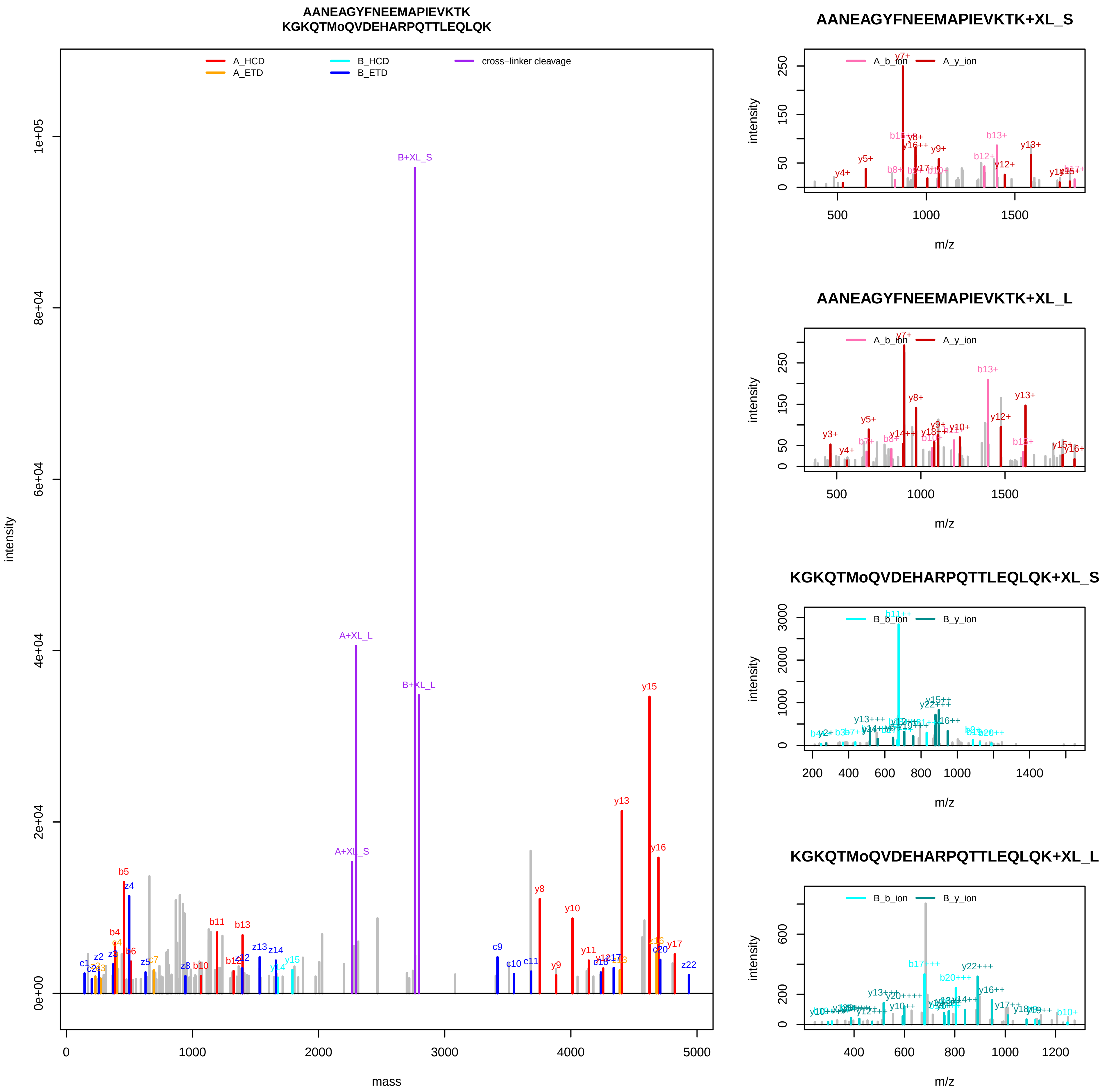
AANEAGYFNEEMAPIEVKTK
KGKQTMoQVDEHARPQTTLEQLQK
A_HCD
A_ETD
B_HCD
B_ETD
cross−linker cleavage
intensity
mass
B+XL_S
A+XL_L
B+XL_L
A+XL_S
y15
y13
y16
y17
y8
y10
y11
y9
b5
z4
b4
b6
b11
b13
b10
z13
z14
z8
z5
z3
z2
c1
c9
c10
c11
c16
c20
z22
0e+00
2e+04
4e+04
6e+04
8e+04
1e+05
0
1000
2000
3000
4000
5000
AANEAGYFNEEMAPIEVKTK+XL_S
A_b_ion
A_y_ion
y7+
b13+
y13+
y5+
y4+
y9+
y12+
intensity
m/z
AANEAGYFNEEMAPIEVKTK+XL_L
y3+
y8+
y10+
y16+
KGKQTMoQVDEHARPQTTLEQLQK+XL_S
B_b_ion
B_y_ion
b11++
y15++
y22+++
y13+++
KGKQTMoQVDEHARPQTTLEQLQK+XL_L
b17+++
y22+++
b20+++
y16++
y17++
b10+
